# The Functional Roles of *Lactobacillus acidophilus* in Different Physiological and Pathological Processes

**DOI:** 10.4014/jmb.2205.05041

**Published:** 2022-08-30

**Authors:** Huijuan Gao, Xin Li, Xiatian Chen, Deng Hai, Chuang Wei, Lei Zhang, Peifeng Li

**Affiliations:** 1 Institute for Translational Medicine, The Affiliated Hospital of Qingdao University, Qingdao University, 308 Ningxia Road, Qingdao 266071, P.R. China; 2 Department of Chemistry, University of Aberdeen, Aberdeen, AB243UE, UK

**Keywords:** Probiotics, *Lactobacillus acidophilus*, intestinal flora, cholesterol, immunity

## Abstract

Probiotics are live microorganisms that can be consumed by humans in amounts sufficient to offer health-promoting effects. Owing to their various biological functions, probiotics are widely used in biological engineering, industry and agriculture, food safety, and the life and health fields. *Lactobacillus acidophilus* (*L. acidophilus*), an important human intestinal probiotic, was originally isolated from the human gastrointestinal tract and its functions have been widely studied ever since it was named in 1900. *L. acidophilus* has been found to play important roles in many aspects of human health. Due to its good resistance against acid and bile salts, it has broad application prospects in functional, edible probiotic preparations. In this review, we explore the basic characteristics and biological functions of *L. acidophilus* based on the research progress made thus far worldwide. Various problems to be solved regarding the applications of probiotic products and their future development are also discussed.

## Introduction

The Food and Agriculture Association (FAO) and World Health Organization (WHO) have highlighted that probiotics are live strains of microorganisms that have been carefully selected. When administered in sufficient amounts, probiotics can bring health benefits to the host [1]. There are a large number of probiotic bacteria that colonize the human intestine, which can also interact and co-evolve with the human body. They help the host to digest and absorb nutrients in food, metabolize toxic waste products, and produce amino acids and short-chain fatty acids necessary for normal human activities. They can provide definite health effects such as improving the host microecological balance while exerting other beneficial effects on the intestinal tract [2].

Since the early 1990s, a plethora of "probiotic" health products have swept throughout the global market. In the meantime, "probiotics" have become a hot international research topic. Probiotics have been extensively studied in a variety of diseases and have been demonstrated to produce a range of potential health effects. The most studied species include lactobacilli, bifidobacteria, and yeasts [3]. Among them, *L. acidophilus*, an important intestinal probiotic in the lactic acid bacteria (LAB) family, has had a great deal of focus placed upon it in terms of research and development, especially as it is so closely linked to human health. As such, *L. acidophilus* is widely considered to have probiotic effects and is one of the most commonly recommended microorganisms for dietary use [4]. Compared with many other probiotics, *L. acidophilus* has better resistance to both acid and bile salt. These characteristics facilitate the survival and proliferation of *L. acidophilus* in the harsh environment of the gastrointestinal tract. Its ability to survive under these conditions provides further opportunities for its products to successfully function in the human body. When the total amount of *L. acidophilus* reaches a certain threshold value, health promotion can be achieved. *L. acidophilus* has multiple effects on the human body, including nutritional effects, regulation of intestinal flora balance, enhancement of immunity, age-delaying and anti-cancer effects, and support of cholesterol reduction [5, 6].

In this review, the basic characteristics of *L. acidophilus* are summarized, its biological functions in various diseases are discussed, and future research directions and applications in the form of probiotic products are explored. We hope to unveil the relationships between *L. acidophilus* and various life activities and disease development so as to provide a theoretical basis for later research and application direction.

## Basic Features and Functional Mechanisms of *L. acidophilus*

*L. acidophilus* was initially isolated from infant feces in 1900 and was officially named *Lactobacillus acidophilus*. Subsequently, a series of biological characteristics and functions have been studied. *L. acidophilus* is subdivided into many strain types, including *L. acidophilus* LA-1, LA-5, NCFM, and ATCC4356, DDS-1 [7]. Different strains also confer differing probiotic properties and functions. Since a strain may have multiple names, it is easy to confuse the strains during the process of understanding them and when undertaking research. Fortunately, most of the work on *L. acidophilus*, especially the work relating to its probiotic effects, has been done on particular strains.

### Basic Features of *L. acidophilus*

*L. acidophilus*, within the genus *Lactobacillus* in the family *Lactobacillaceae*, is a gram-positive bacillus that does not form spores. They present as slender rods with a circular end, measuring 2-10 μm long. Most *L. acidophilus* strains are microaerobic bacteria, which grow better in anaerobic environments, or in 5~10% CO_2_, rather than in aerobic environments. Their optimum culture temperature is generally 35~38°C, and they basically do not grow in temperatures below 20°C. *L. acidophilus* has poor heat resistance and its optimum pH is 5.5~6.0. The growth characteristics of different strains are also slightly different from each other. *L. acidophilus* is eosinophilic and has good resistance toward acids and bile [8, 9]. It can grow and reproduce in environments where other LAB cannot grow. It can also use glucose, fructose, lactose and sucrose to carry out homotype fermentation and can produce DL-lactic acid via fermentation [10].

**Fig. 1:**
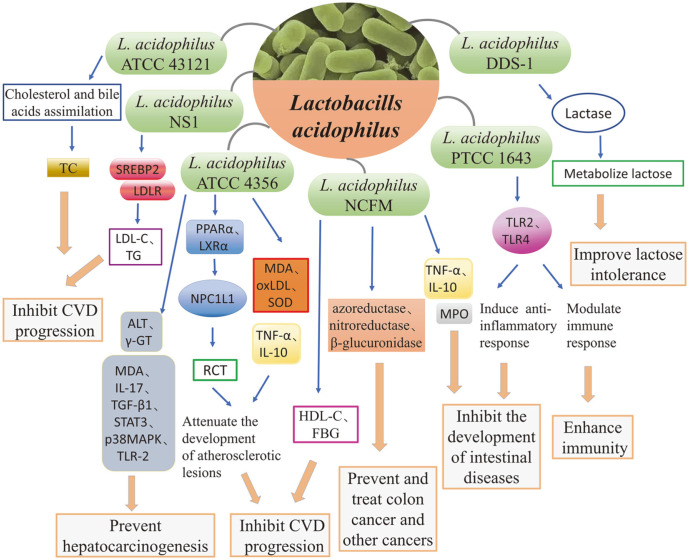
Probiotic properties and biological functions of *Lactobacillus acidophilus*. *L. acidophilus* is a species of beneficial microbial flora and has been proven to play an important role in many pathological and physiological processes. It has been shown to improve CVD and lactose intolerance, prevent and treat cancer, regulate immunity, and improve gastrointestinal diseases.

*L. acidophilus* is a species of beneficial microbial flora and has been proven to have many good probiotic characteristics that can be roughly divided into two categories. The first category covers the essential probiotic properties of *L. acidophilus* which have been demonstrated in vitro and include tolerance to low pH, bile resistance, adhesion to human colon cells in cell culture, antibiotic production, lactase activity, and product stability [11-17]. The second category includes the overall probiotic effects that have been observed in animal-level feeding studies, such as regulation of host immune responses, reduction of host serum cholesterol, improvement of host lactose metabolism, and prevention or treatment of infection [18-20]. Here, we summarize the basic probiotic properties and main biological functions of *L. acidophilus* (Fig. 1).

### Functional Mechanisms of *L. acidophilus*

According to current studies, *L. acidophilus* participates in the host intestinal tract mainly through the production of metabolites and regulation of intestinal microbiota [21, 22].

First, *L. acidophilus* can regulate the balance of intestinal flora by reducing the intestinal pH and producing metabolites [21, 23]. The optimal pH of many intestinal pathogenic bacteria is neutral or slightly alkaline. Lactic acid produced by *L. acidophilus* metabolism can reduce pH, thus inhibiting the growth and reproduction of pathogenic bacteria [23]. In addition, some pathogenic microorganisms produce enzymes that can catalyze the conversion of carcinogenic precursors to carcinogens, such as azo reductase, nitro reductase, and β-glucosidase [24, 25]. *L. acidophilus* can not only inhibit the growth of these pathogenic microorganisms and reduce the production of these enzymes, but can also inhibit enzyme activity [25, 26]. Second, competition for adhesion sites with pathogenic bacteria is an important mechanism for *L. acidophilus* to inhibit the function of pathogenic bacteria, thereby interfering with their invasion into cells [27]. Surface the S-layer protein, extracellular polysaccharide and lipoteichoic acid of many strains can compete for adhesion with pathogenic bacteria [21, 28]. Third, the role of *L. acidophilus* in many diseases also depends on its ability to reduce serum cholesterol level [29, 30]. *L. acidophilus* can absorb and assimilate cholesterol [31, 32].

Although some mechanisms have been found, neither these mechanisms nor the influencing factors of *L. acidophilus* in the host have been fully studied and efforts should be made to explore them further.

## Biological Functions of *L. acidophilus*

### Risk Reduction of Cardiovascular Disease

Cardiovascular disease (CVD) is the leading cause of morbidity and mortality worldwide, accounting for about one third of global deaths [33]. The occurrence of CVD is related to many factors [34-36]. Increasing blood cholesterol is one risk factor that directly impacts CVD. In the 1970s, Mann and Shaper found that populations of particular African tribes generally possessed lower incidences of high serum cholesterol [37]. After investigation, they found that the residents of these tribes regularly drank yogurt fermented by *L. acidophilus*, suggesting that this bacteria might regulate blood lipids [37]. Since then, more researchers have paid attention to the uses of *L. acidophilus* and other probiotics relevant to cholesterol reduction and the cholesterol-lowering effects of *L. acidophilus* have been confirmed. In addition, studies have investigated the effects of *L. acidophilus* intervention on cholesterol reduction and atherosclerosis development in animal models.

Harrison and Peat reported that the addition of *L. acidophilus* to baby feed reduced infant serum cholesterol from 147 mg/100 ml on the 5th day down to 119 mg/100 ml by the 8th day of intervention [38]. This decrease in serum cholesterol levels was accompanied by a significant increase in the number of LAB. The number of *Escherichia coli* present in fecal samples also decreased [38]. Stepankova *et al*. found that supplementation of *L. acidophilus* ATCC 4356 also promoted the proliferation of bifidobacteria [39]. Gilliland and Walker showed that *L. acidophilus* NCFM was able to remove cholesterol from laboratory growth media [40, 41]. NCFM has been reported to ingest cholesterol in the presence of bile and in the absence of oxygen, both of which occur in the gut. The researchers tested these effects on young pigs and the results showed that feeding *L. acidophilus* to pigs significantly inhibited the increased serum cholesterol levels that are usually observed in individuals fed a high-cholesterol diet. Park and colleagues have reported that adding *L. acidophilus* 43121 to a high-cholesterol diet given to rats resulted in reduced serum cholesterol [42, 43]. Song *et al*. showed that *L. acidophilus* NS1 can reduce plasma LDL-C by increasing the expression of LDLR and SREBP2 in the liver [44].

Huang et al. found that *L. acidophilus* ATCC 4356 had a significant cholesterol-lowering effect on rats fed with a high-cholesterol diet by inhibiting the expression of NPC1L1 in the small intestine [31, 45]. Chen and colleagues found that *L. acidophilus* ATCC 4356 alleviated atherosclerotic lesions in *ApoE^−/−^* mice[46]. *L. acidophilus* ATCC 4356 inhibited oxidative stress by regulating the production of MDA, oxLDL and SOD, suppressed inflammation via regulations of TNF-α and IL-10 levels, and improved intestinal flora, resulting in blocked progression of atherosclerosis. However, it did not significantly reduce cholesterol levels. In different experimental models, many studies have produced different results due to inconsistent experimental methods. Therefore, the role and functional mechanism of *L. acidophilus* in reducing cholesterol and alleviating atherosclerosis require further detailed exploration.

### Improvement of Gastrointestinal Disease Outcomes

Studies have shown that probiotics can regulate intestinal flora, play a beneficial role in inflammatory diseases such as ulcerative colitis (UC), and have been used effectively to treat and suppress human intestinal infections. Lightfoot and colleagues described the role of *L. acidophilus* NCFM surface layer protein A as a key effector in the prevention of colitis in mice [47] . Chandhni *et al*. also showed that the surface proteins in NCFM strains could reverse histopathological damage caused by colitis [48], thus providing a potentially safer option for the treatment of inflammatory bowel disease.

In the normal intestinal flora in humans, *L. acidophilus* plays a key role in inhibiting the growth of pathogens such as *Salmonella enteritidis*, *Staphylococcus aureus*, and *Shigella dysenteriae*. Therefore, studies have shown that *L. acidophilus* exhibited strong anti-inflammatory activities [49]. Moshiri *et al*. reported that *L. acidophilus* PTCC 1643 could affect the expression of TLR2 and TLR4 in HT29 intestinal epithelial cells under the action of *Salmonella enterica* serovar Enteritidis (SesE), and inhibit the inflammatory response caused by SesE infection [50]. Small intestinal bacterial overgrowth (SIBO) refers to the changes in the number or types of flora in the small intestine. It is considered to be a condition that can exist for many years without causing obvious symptoms although it is related to chronic digestive problems. Studies by Simenhoff and colleagues have shown that NCFM can inhibit the overgrowth of small intestinal bacteria, reduce the levels of toxic metabolites such as dimethylamine and nitrosodimethylamine in the blood, and positively affect intestinal colonization [51, 52], thus improving the nutritional status of patients. These observations support the use of *L. acidophilus* for the prevention and treatment of intestinal diseases. Further in vivo and in vitro studies are needed to elucidate the detailed mechanisms of these anti-inflammatory effects.

### Improvement of Lactose Intolerance

Lactose intolerance, also known as lactose indigestion or lactose malabsorption, refers to the state in which the human body does not produce the enzyme lactase. After consuming milk or dairy products, some people might have diarrhea and other symptoms of intestinal discomfort due to the osmotic effect of the undecomposed lactose.

Previously, many studies have proved that LAB have the ability to be a source of lactase in the small intestine, which helps people with lactase deficiency to digest lactose. Related fermented dairy products may also enhance lactose tolerance [53].

Some probiotic studies have shown that *L. acidophilus* can improve lactose digestion or symptoms in lactose-intolerant patients [54, 55]. Among these studies, in vitro evaluation of the lactase levels of various probiotics has shown that the lactase levels of *L. acidophilus* NCFM were high when compared to all of the probiotics tested. Multiple studies have also shown that NCFM can improve lactose digestion and relieve symptoms of lactose intolerance such as bloating and diarrhea [56, 57]. A study speculated that the bacteria might metabolize lactose during digestion and transport it through the gastrointestinal tract. The study by Pakdaman *et al*. found that *L. acidophilus* DDS-1, a unique and edible strain, can improve lactose intolerance symptoms such as diarrhea, cramps, and vomiting [58]. However, a number of studies have shown the opposite effects. For example, Newcomer *et al*. demonstrated that dairy products containing *L. acidophilus* NCFM did not significantly improve human lactose intolerance [59-61]. The reason for these contradictory results has been associated with the levels of NCFM of *L. acidophilus*. Therefore, in order to better apply the functional properties of *L. acidophilus* and to improve lactose intolerance, it is essential to explore and adjust the probiotics levels and the formula with each product.

### Prevention and Treatment of Cancer

Probiotics are considered a safe and cost-effective way to prevent or treat a variety of cancers, including colon and liver cancer. Several studies have suggested that consumption of cultured dairy products may reduce colon cancer risk, since the effects of diet are mediated by metabolic effects of intestinal organisms. The activities of β-glucuronase, nitroreductase, azoreductase and other microbial enzymes have been used to monitor colon cancer changes. Goldin and Gorbach observed that adding live *L. acidophilus* into the diet of carnivorous rats significantly reduced azoreductase, nitroreductase and glucuronidase activity [24, 25]. The incidence of colon cancer in rats with *L. acidophilus* NCFM was also lower. Their later study found that NCFM alongside antibiotics inhibited the growth of colon tumors in rats. In human, daily consumption of milk containing NCFM reduced the activity of these three fecal enzymes by a factor of two- to four-fold and reduced the incidence of colon cancer [24, 25]. In addition, they found that nitroreductase activity continued to decrease even three weeks after fermented milk intake was stopped, thus indicating a long-term change in colonic flora [24, 25].

Studies have shown that the extracellular polysaccharides (EPSs) synthesized by *L. acidophilus* have exerted health benefits by stimulating the immune response and fighting tumor cells [62]. The anticancer and immunomodulatory activities of EPSs synthesized by *L. acidophilus* have been proven to combat colon cancer and inflammatory liver cancer. Khedr and colleagues used male rats as a model and confirmed that *L. acidophilus* ATCC 4356 EPSs had immunomodulatory effects on liver cancer induced by diethylnitrosamine (DEN) and gamma radiation (IR) [63]. They proposed that *L. acidophilus* ATCC 4356 EPSs might be used as a safe and effective probiotic to prevent and treat liver cancer.

### Regulation of Immune Capacity

The immune function of *L. acidophilus* is mainly conducted via regulating the body’s immune system, limiting pathogen colonization within the body, and controlling metabolic disorders and enteritis. Probiotics are used clinically to treat diseases caused by immune system disorders. This can significantly reduce infection time and respiratory tract infection frequency and also improve the therapeutic effects for allergic asthma.

The role of *L. acidophilus* in regulating the body's ability to respond to immune responses has been demonstrated in previous studies. Wagner *et al*. confirmed that NCFM induced antibody- and cell-mediated responses to *Candida albicans* in immunodeficient mice [64]. The serum levels of IgG, IgA, and IgM were higher in euthymic immunocompromised mice and were thought to reduce the severity of candidiasis. Yoghurt prepared with yoghurt cultures containing NCFM, *Streptococcus thermophilus*, *Lactobacillus bulgaricus* and Bifidobacterium were tested for their effects on mucosal and systemic IgA and IgG responses in mice immunized orally with cholera toxin. The results showed that IgA against the cholera toxin was higher in the intestine and serum of the mice fed the formulated yogurt than that observed in the mice fed skim milk [65]. These results suggest that coculture including NCFM may increase immune responses to oral antigens.

### Other Functions

We summarized the biological functions, processes, and effects of *L. acidophilus*- related strains in pathological and physiological processes (Table 1). However, the biological functions of *L. acidophilus* are far more extensive than what we have mentioned. The role of *L. acidophilus* in many other diseases is constantly being explored and new discoveries are being made. Studies have shown that kidney tissue damage can be alleviated by reducing oxidative stress, inflammation, and cell death [66]. Zhang *et al*. first explored the relationship between ATCC 4356 and renal ischemia-reperfusion injury (IRI) [67]. They found that *L. acidophilus* ATCC 4356 alleviated renal IRI through antioxidant stress and anti-inflammatory responses and improved intestinal microbial distribution in renal IRI mice. In the following treatment with ATCC 4356, the levels of anti-inflammatory factors (IL-4 and IL10) were upregulated, whereas the levels of pro-inflammatory factors (IL-1β, IL-8, TNF-α and IFN-γ) were downregulated. In addition, renal tissue apoptosis in IRI mice was reduced [67].

Studies have shown that oral *L. acidophilus* can improve heart function in mice with myocardial infarction. Sadeghzadeh and co-researchers found that *L. acidophilus* was able to improve the hemodynamic and histopathological indicators of the ISO-induced myocardial injury rat model [68], providing obvious myocardial protection. In the future, probiotic supplements may become a new option for patients with ischemic heart disease.

*L. acidophilus* has been shown to have potential applications in the prevention and control of genitourinary and vaginal infections. Reid *et al*. precultured *L. acidophilus* NCFM with urinary and vaginal epithelial cells from healthy women and subsequently exposed them to different urinary tract pathogens. Results showed that NCFM competitively excluded these pathogens and effectively prevented and suppressed urinary tract and vaginal infections [69].

Rheumatoid arthritis (RA) is a common inflammatory joint disease. It has been reported that the ingestion of *L. acidophilus* ATCC 314 exerted anti-inflammatory and potent antioxidant properties in a collagen-induced arthritis (CIA) rat model [70, 71]. This suggests that *L. acidophilus* is a promising treatment that should be tested further in RA patient preclinical trials.

## Applications and Future Prospects of *L. acidophilus*

As people pay more and more attention to health issues, it is of great importance that different kinds of probiotics within food are able to play a healthy role. Of these probiotics, *L. acidophilus* is one of the most commonly used microorganisms, as it is thought to have various beneficial effects on human health. These advantageous effects include lowering blood cholesterol, improving gastrointestinal diseases, and reducing the risk of lactose intolerance and carcinogenicity [72, 73]. Research and development into *L. acidophilus* has received widespread attention. It is referred to as the third-generation yogurt starter strain. *L. acidophilus* has good acid and bile salt resistance and produces a variety of antibacterial substances during metabolism. Due to these attributes, *L. acidophilus* strains have broad application prospects as functional, edible bacteria.

Despite these favorable characteristics, through our analysis of studies on *L. acidophilus* which highlighted our present understanding of the current application status, we found that there are also many problems and limitations in the research and application of *L. acidophilus*. First, due to different naming methods, the same strain may have multiple names. This may lead to confusion during literature searches and research. Important findings could be missed, resulting in incomplete information collection. Second, due to the different research methods used to investigate probiotics, results from different research groups may deviate from each other. Different concentrations and ratios of probiotics have been shown to have different effects, therefore the health benefits of certain disease symptoms remain to be proven. Further research on the optimal strains, doses and dosing algorithms are of key importance for future research. In addition, different species of *L. acidophilus* can exhibit similar probiotic effects in vitro, but their properties differ significantly when evaluated in vivo.

Currently, probiotic regulation of intestinal flora is recognized as an interesting way to prevent certain diseases. Recent studies have proposed many mechanisms by which probiotics function, but the effectiveness of many probiotics has not been proven in different conditions, which has presently limited the promotion and application of probiotics. There are probably several main reasons why these research limitations occur, including too many low-quality studies, variability within the microbiome, and great diversity between the probiotic strains used. However, some studies have reported reasonable and encouraging results that support further research into probiotics. With this in mind, we should put more effort into overcoming the difficulties. First, because most studies have, so far, focused on animal studies or small human research groups, it is difficult to assess the possible health effects of these probiotics in the general population. Therefore, we need to conduct more extensive epidemiological evaluations which take into account the variability between patients. Due to the high cost of such interventions, it is necessary to characterize strains well to select the strains that are most effective for a particular application. Second, we should identify bacterial markers of the microbiome in related diseases in order to gain sufficient clinical trial capacity. Furthermore, new methods for analyzing the microbiome and its function will greatly facilitate the research when studying large numbers of samples. Probiotics could serve as a low-cost, low-risk alternative to antibiotic treatment in order to prevent infection. We believe that the development of probiotics will open up another impressive field of research. Further research in this area may provide exciting avenues for healthcare strategies, as well as creating more economic and social benefits.

## Figures and Tables

**Table 1:** The main biological effects of related strains of *L. acidophilus*.

Type of strain	Function	Major biological processes and their effects	Reference
*L. acidophilus* ATCC 4356	Inhibit CVD progression	Inhibit oxidative stress by modulating the productions of MDA, oxLDL and SOD; suppress inflammatory status by regulating TNF-α and IL-10 levels; inhibit NPC1L1 expression in the small intestine; improve intestinal microflora; inhibit the development of atherosclerosis.	[31, 40, 45, 46]
*L. acidophilus* NCFM		Assimilate cholesterol and control cholesterol levels.	[40, 41]
*L. acidophilus* 43121		Affect cholesterol metabolism and reduce blood cholesterol levels.	[40, 42, 43]
*L. acidophilus* NS1		Reduce plasma LDL-C by increasing hepatic LDLR and SREBP2 expression.	[44]
*L. acidophilus* NCFM	Improve gastrointestinal diseases	Strain surface layer proteins play an important role; alleviate Tcell-induced colitis by significantly reducing the proinflammatory response; preserve microbiome composition and intestinal barrier function; reverse histopathological damage caused by colitis; reduce the level of toxic metabolites.	[47, 48, 51, 52]
*L. acidophilus* PTCC 1643		Modulate the expression of TLR2 and TLR4 in HT29 intestinal epithelial cells challenged with SesE; enhance anti-inflammatory effects.	[50]
*L. acidophilus* NCFM	Improve lactose intolerance	Strain has a higher level of lactase, which metabolizes lactose during digestion and transits through the gastrointestinal tract, thereby improving lactose digestion.	[56, 57]
*L. acidophilus* DDS-1		Assist in breaking down lactose; improve lactose intolerance symptoms such as diarrhea, cramps and vomiting.	[58]
*L. acidophilus* ATCC 4356	Prevent and treat colon cancer, liver cancer and other cancers	The exopolysaccharides of the strain have immunomodulatory and antitumor activities; regulate the TLR2/STAT-3/P38-MAPK pathway associated with inflammation against HCC.	[63]
*L. acidophilus* NCFM		Stimulate the immune response; reduce the activities of β-glucuronase, nitroreductase, azoreductase and other microbial enzymes; produce compounds that inhibit tumor proliferation; reduce the incidence of colon cancer and inhibit the growth of colon tumors.	[24, 25]
*L. acidophilus* NCFM	Regulate immune capacity	Reduce levels of pro-inflammatory cytokines significantly and mobilize a systemic immune response; limit pathogen colonization in the body, control metabolic disorders.	[47, 64, 65]
*L. acidophilus* ATCC314	Manage inflammatory disorders	Regulate the secretion of inflammatory cytokines; reduce oxidative stress.	[70]
